# Supplementary figures and images for: Human cytomegalovirus evades ZAP detection by suppressing CpG dinucleotides in the major immediate early 1 gene
Source: PLoS Pathog. 2020 Sep 4;16(9):e1008844. doi: 10.1371/journal.ppat.1008844 (PMC7498042; doi:10.1371/journal.ppat.1008844)

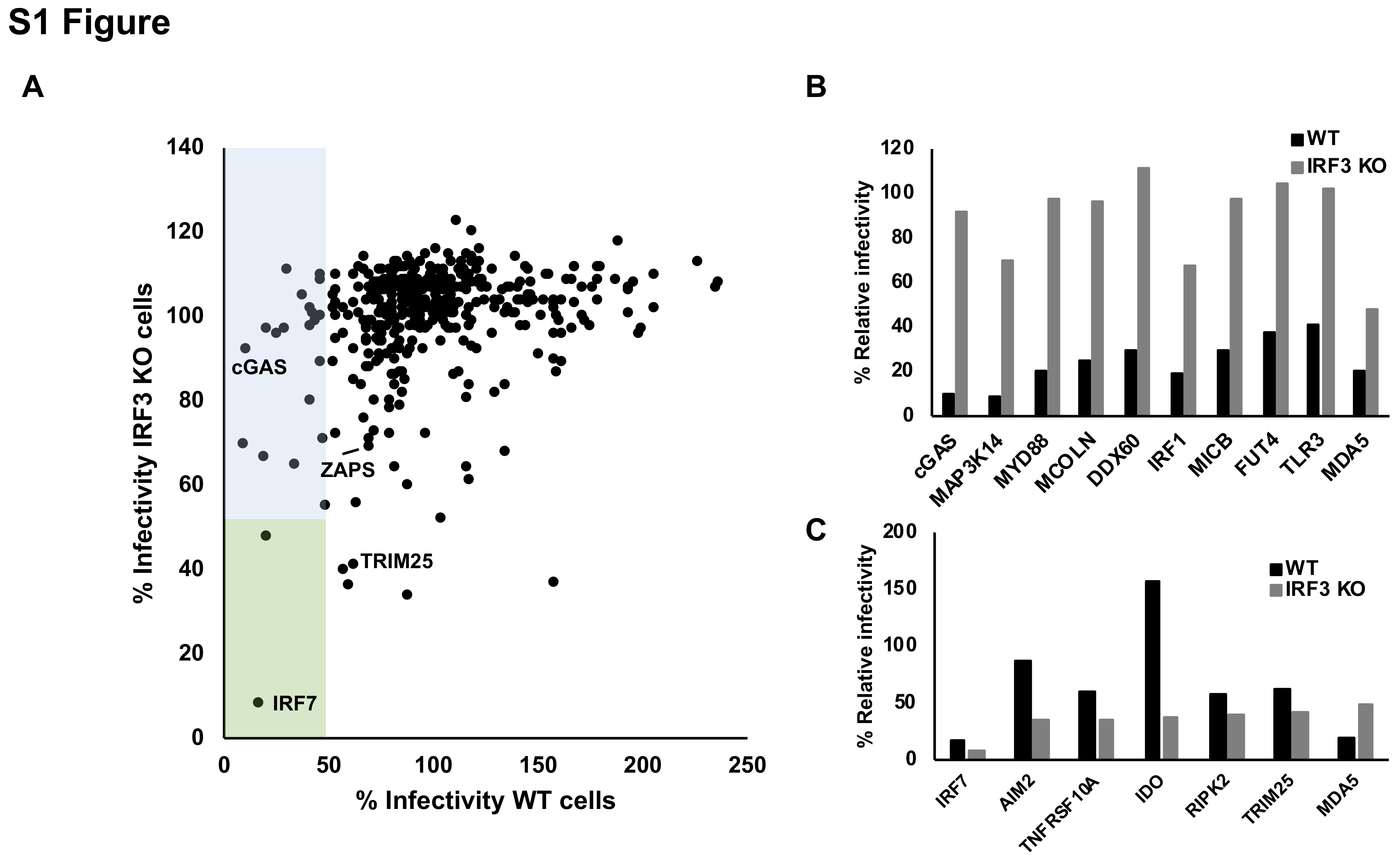

Supplement: S1 Fig — (A) Direct comparison of relative HCMV primary replication for each individual ISG between wild type (WT) and IRF3 knockout (KO) fibroblast cells. Blue box = ISGs that reduced virus production by more than 2-fold in wild type cells, but were not in IRF3 KO cells. The green box = ISGs that reduced virus production by more than 2-fold in both wild type and IRF3 KO cells. Relative primary replication and virus production levels of HCMV for the ISGs in the blue box (B) and for ISGs that reduced primary replication by more than 50% (C) were plotted. (TIF) [file ppat.1008844.s001.tif]

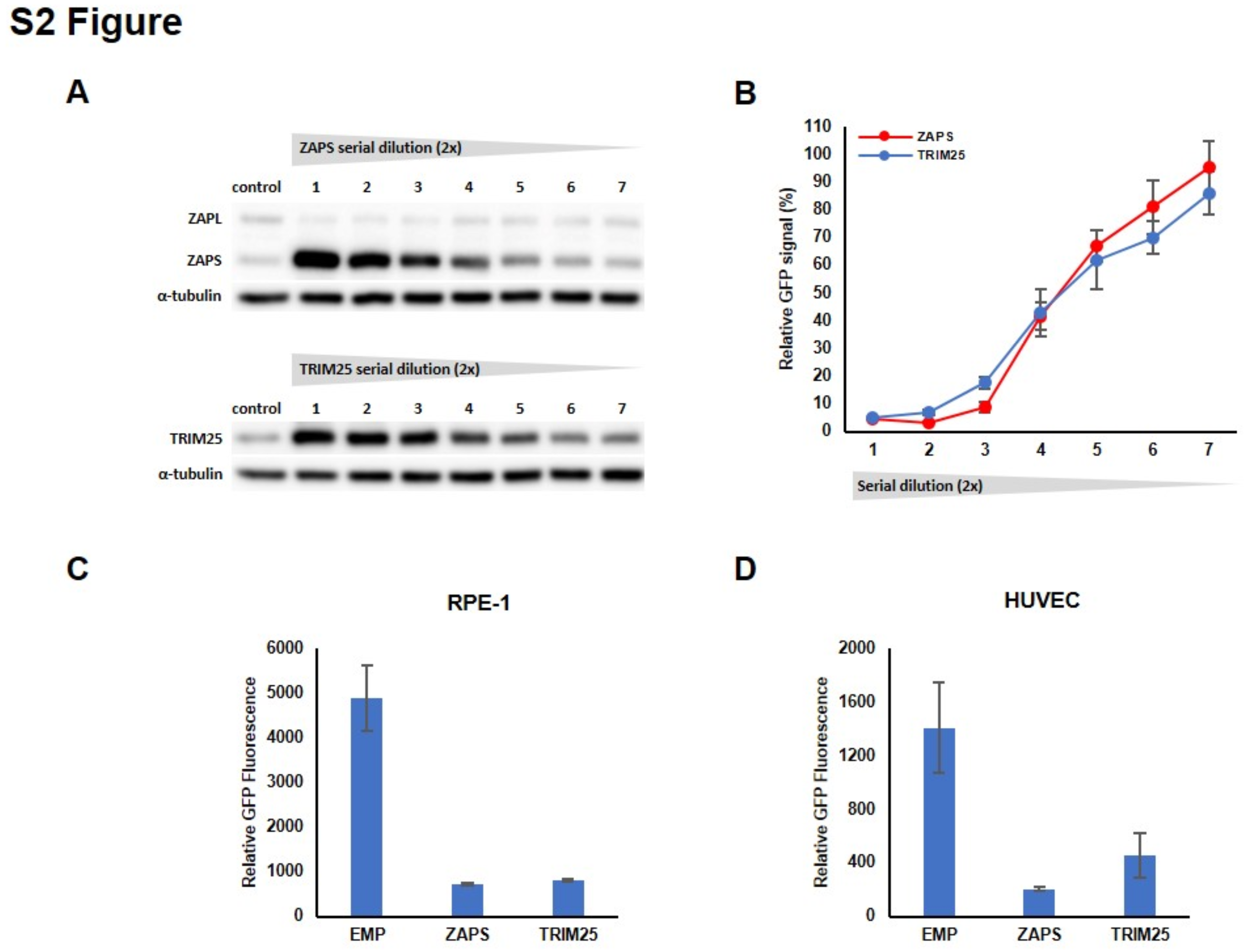

Supplement: S2 Fig — ZAPS and TRIM25 overexpression inhibits HCMV replication (A) Fibroblast cells were transduced with a two-fold dilution series of control empty lentivirus (control) or lentivirus expressing ZAPS or TRIM25. Cells were infected with TB40E-GFP at an MOI of 3 with total protein samples harvested 24 HPI and subjected to western blot analysis for ZAP and TRIM25 levels. (B) GFP levels were measured by plate fluorometry seven days post infection for the corresponding dilution series. GFP levels are shown as a percentage of infected cells transduced with a corresponding serial dilution of a control empty lentivirus (N = 3). Human Epithelial cells (RPE-1) (C) or human umbilical vein endothelial cells (HUVEC) (D) were transduced with a control empty lentivirus (EMP) or lentivirus expressing ZAPS and TRIM25, then infected with TB40E-GFP at an MOI of 3 (titred on corresponding cell line). N = 3. (TIF) [file ppat.1008844.s002.tif]

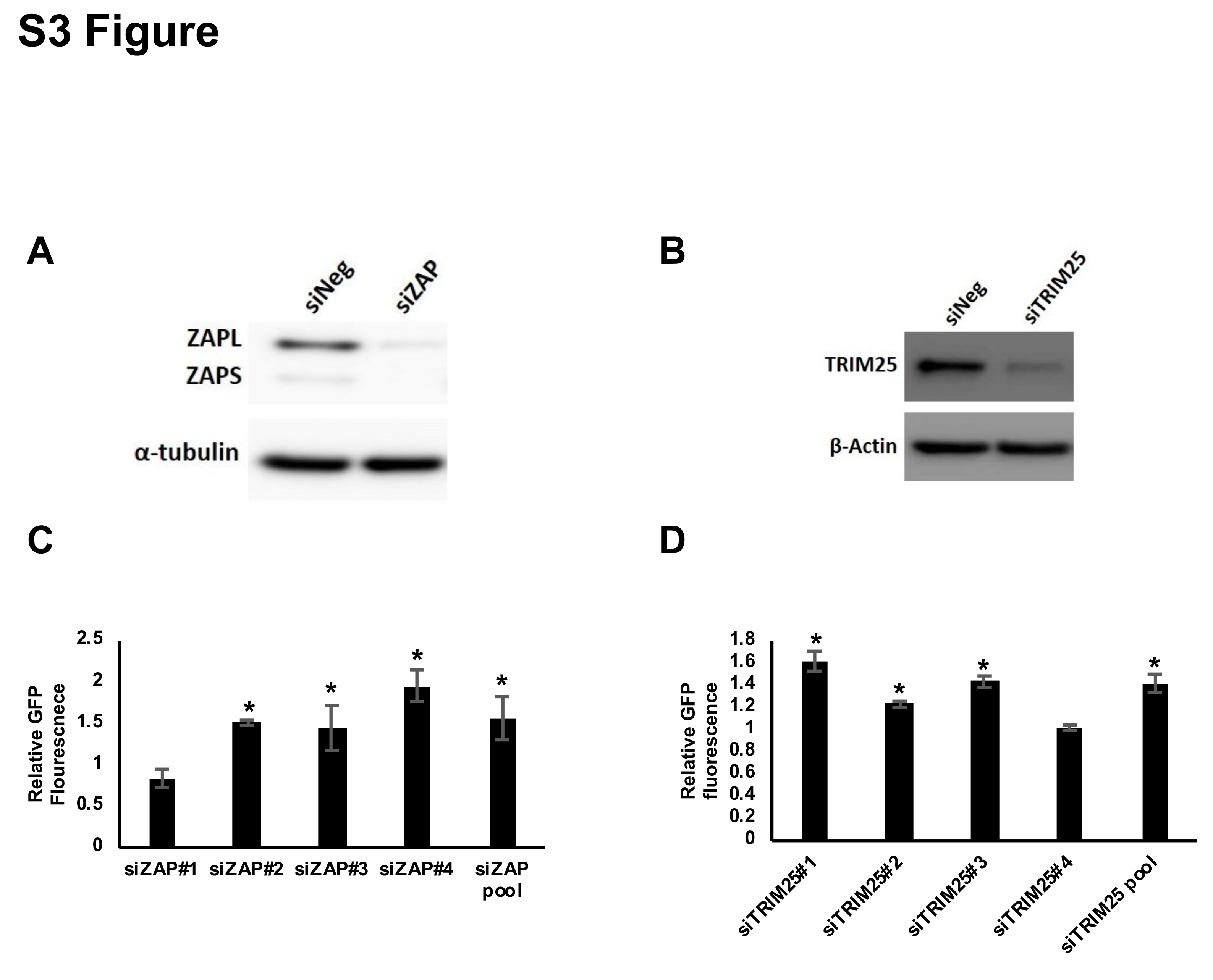

Supplement: S3 Fig — Fibroblast cells were transfected with siRNA targeting ZAP (siZAP), TRIM25 (siTRIM25) or a negative control siRNA (siNeg). Total protein was harvested three days post transfection and levels of ZAP (A) and TRIM25 (B) determined by Western blot analysis. Pools of four siRNAs against ZAP (C) and TRIM25 (D) were deconvoluted and tested individually for effect on HCMV replication. Levels of virus in supernatant were tested seven days post infection with GFP levels normalised to cells transfected with a negative control siRNA. N = 4, * p-value < 0.05 based on Student’s t-test. (TIF) [file ppat.1008844.s003.tif]

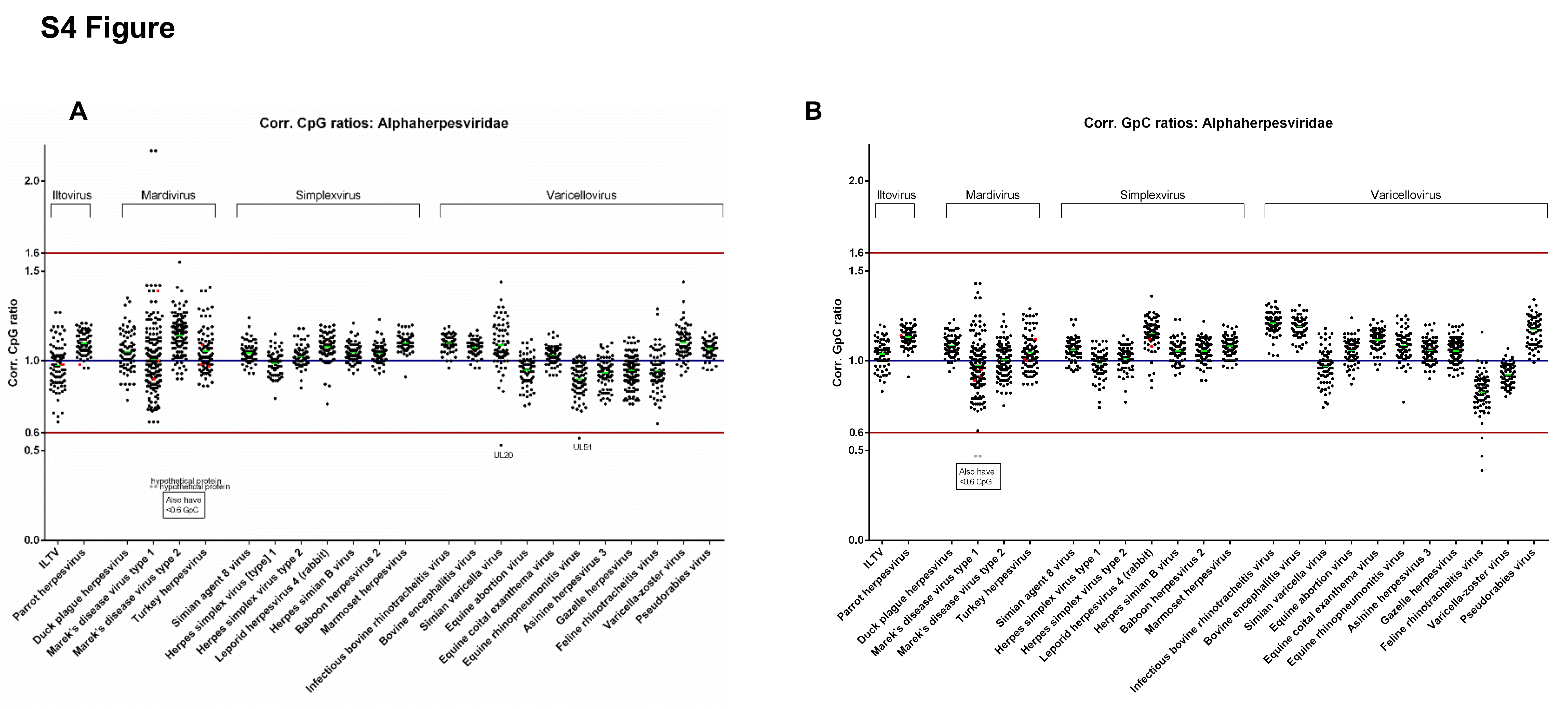

Supplement: S4 Fig — CpG (A) and GpC (B) corrected ratios for ORFs of alpha-herpesviruses. Corrected CpG and GpC ratios were calculated for alpha-herpesviruses as described in the materials and methods and in Fig 3. (TIF) [file ppat.1008844.s004.tif]

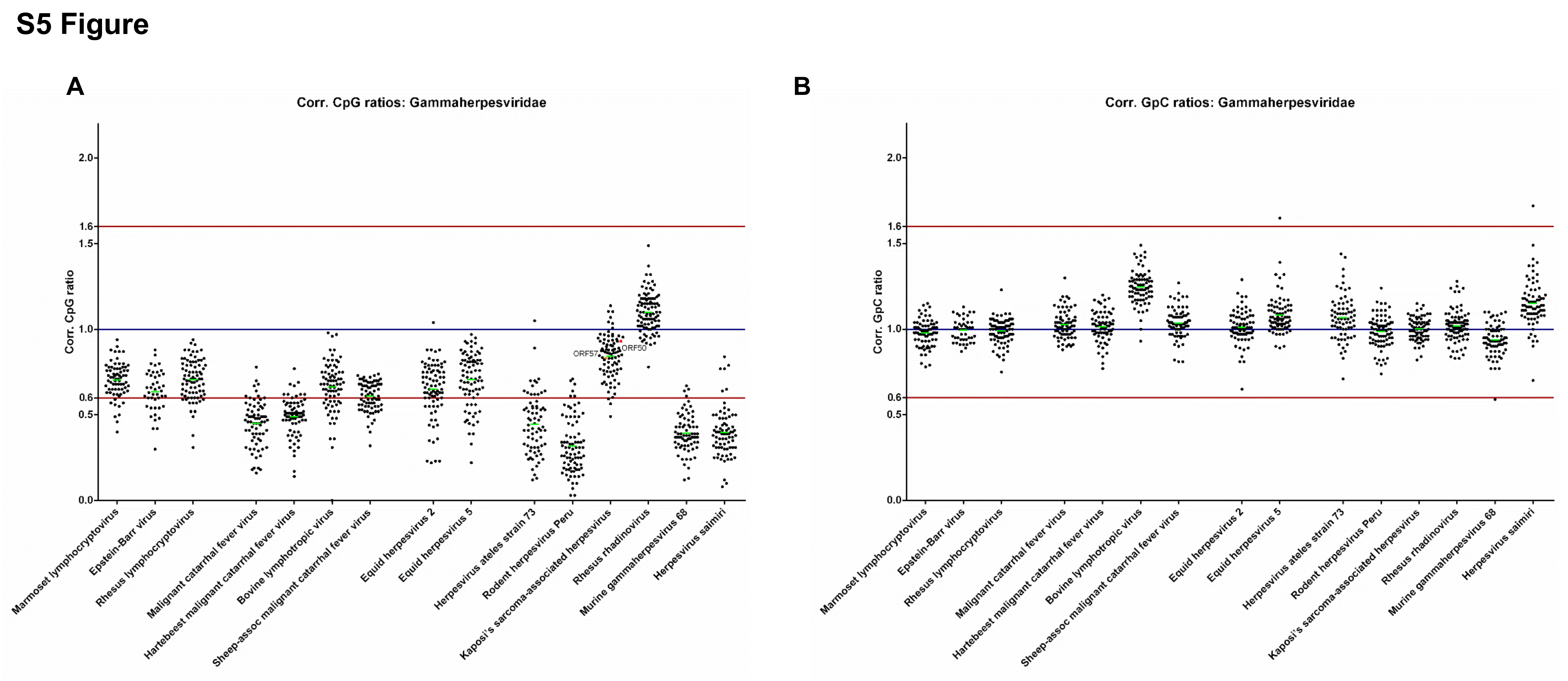

Supplement: S5 Fig — CpG (A) and GpC (B) corrected ratios for ORFs of gamma-herpesviruses. Corrected CpG and GpC ratios were calculated for gamma-herpesviruses as described in the materials and methods and in Fig 3. (TIF) [file ppat.1008844.s005.tif]

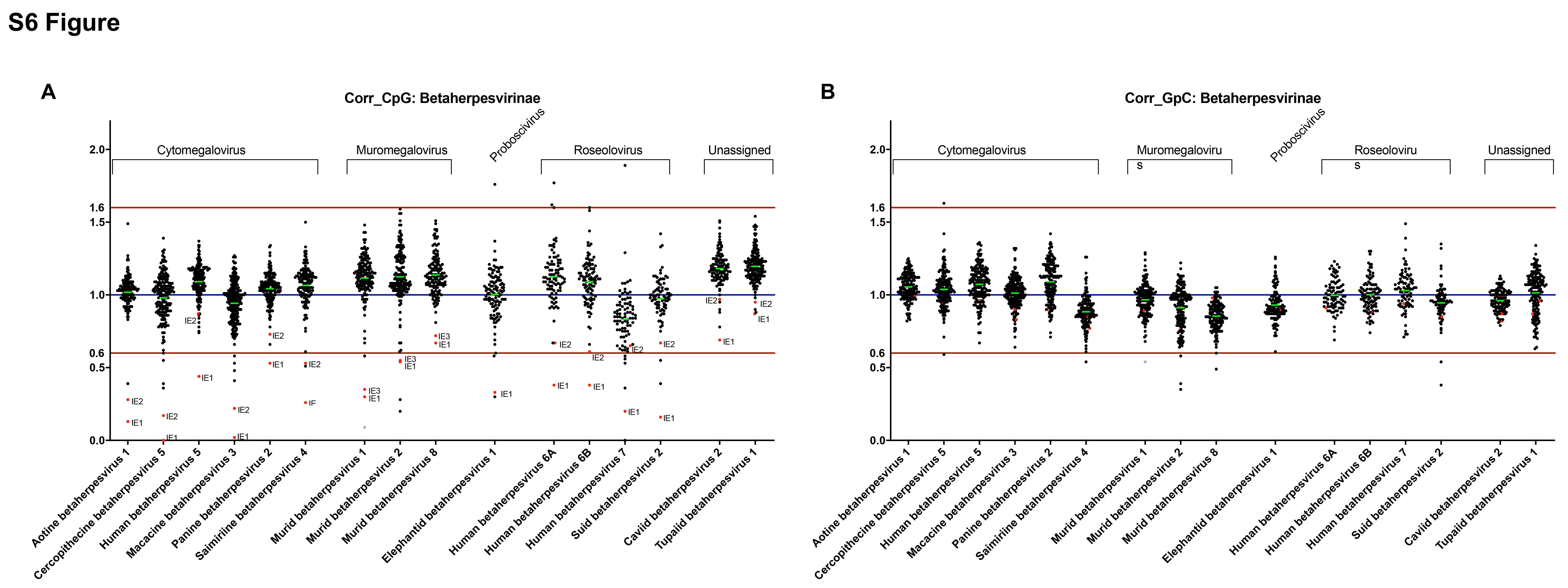

Supplement: S6 Fig — CpG (A) and GpC (B) corrected ratios for ORFs of beta-herpesviruses. Corrected CpG and GpC ratios were calculated for beta-herpesviruses as described in the materials and methods and in Fig 3. (TIF) [file ppat.1008844.s006.tif]

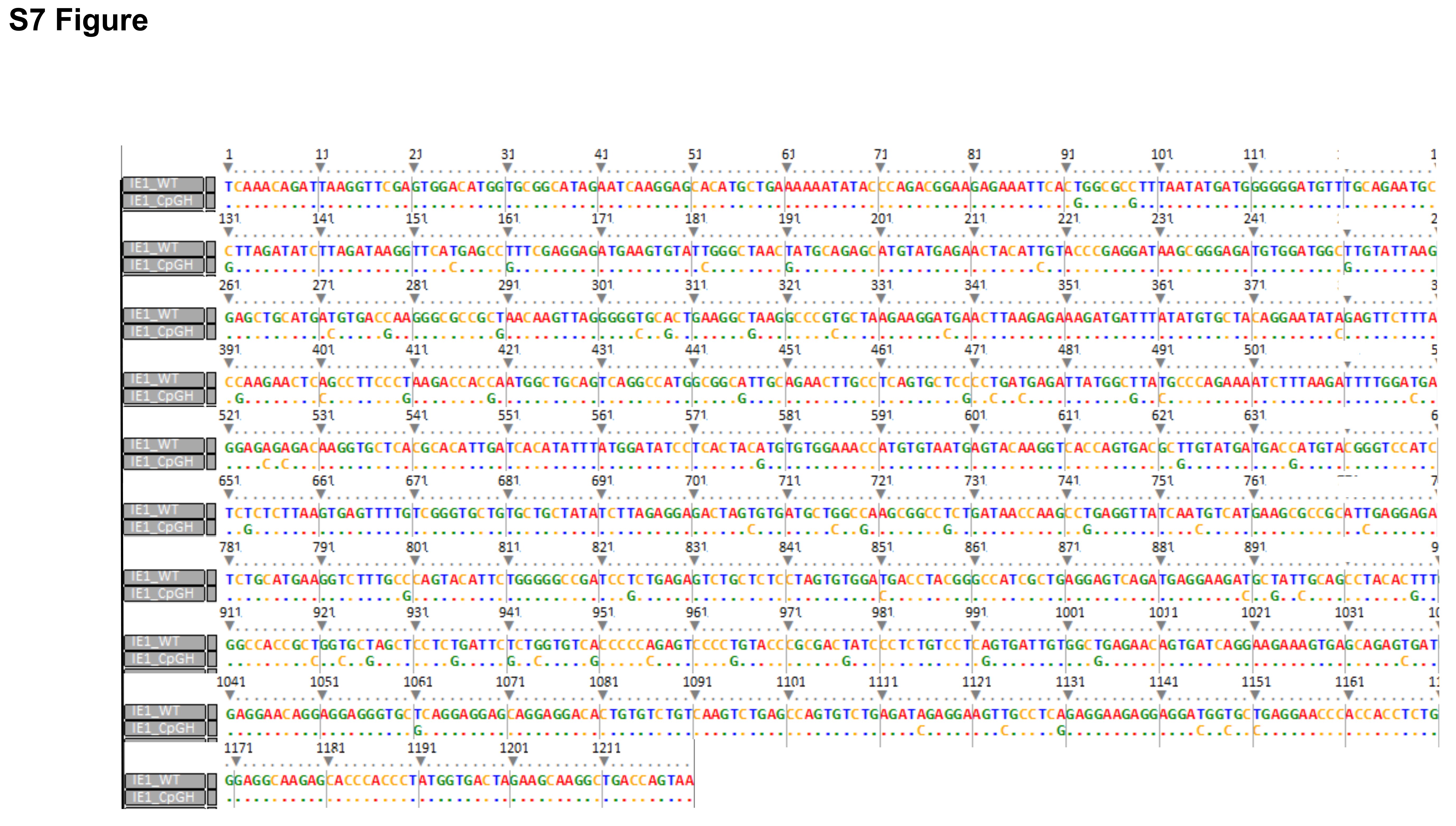

Supplement: S7 Fig — Exon 4 of IE1 was synthesised with synonymous mutations introduced that create CpG motifs. Synonymous mutations indicated below the WT sequence. (TIF) [file ppat.1008844.s007.tif]

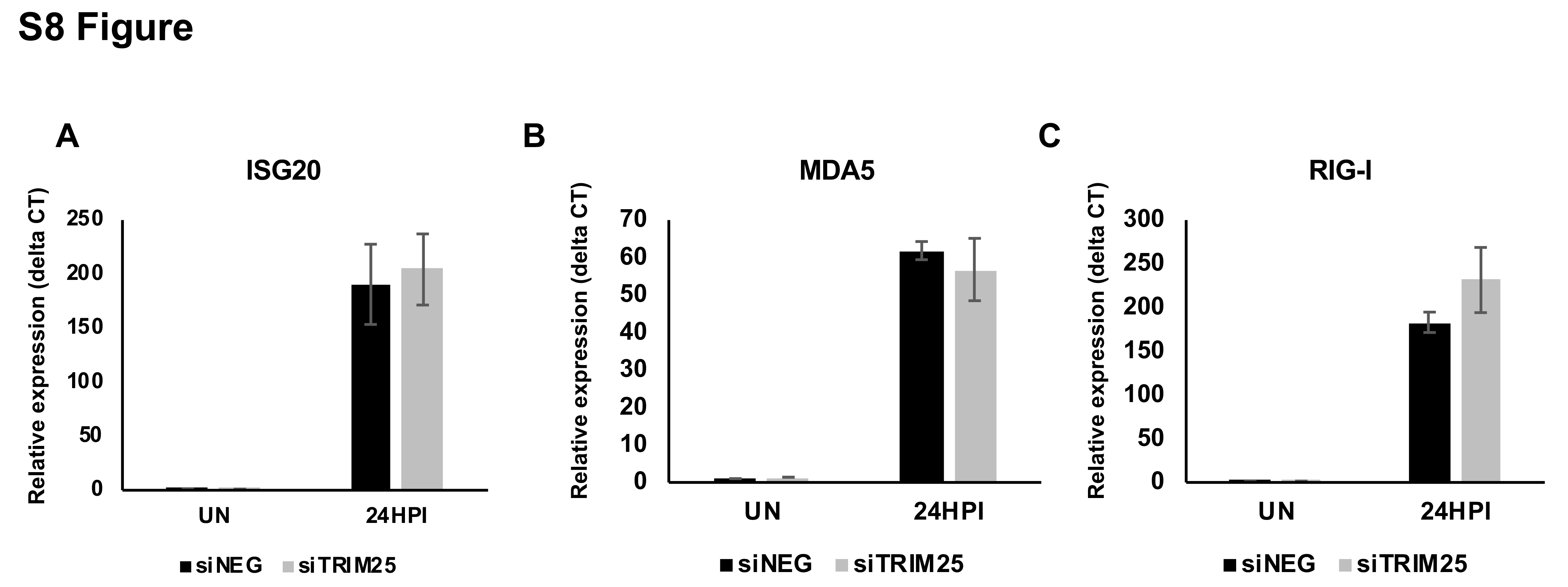

Supplement: S8 Fig — Wild-type fibroblast cells were transfected with TRIM25 siRNA or negative control siRNA (siNEG). 48 hours later, the cells were infected with TB40E-GFP at an MOI of 3. Total RNA was harvested at 24 hours post infection and the RNA levels of (A) ISG20, (B) MDA5 and (C) RIG-I were determined by RT-qPCR analysis and compared to uninfected (UN) cells harvested at the same time point. The result demonstrates that ISG induction following HCMV infection is not inhibited by TRIM25 knockdown. N = 2. (TIF) [file ppat.1008844.s008.tif]
