# Supplementary material for: Human cytomegalovirus evades ZAP detection by suppressing CpG dinucleotides in the major immediate early 1 gene
Source: PLoS Pathog. 2020 Sep 4;16(9):e1008844. doi: 10.1371/journal.ppat.1008844 (PMC7498042; doi:10.1371/journal.ppat.1008844)
Supplement: Attachment — Submitted filename: Reviewer comments HCMV ZAP.docx [file ppat.1008844.s011.docx]

**Part II – Major Issues: Key Experiments Required for Acceptance**
Please use this section to detail the key new experiments or modifications of existing experiments that should be absolutely required to validate study conclusions.

***Reviewer #1:***

*1) Abstract, line 28 and Results, line 127: Is TRIM25 being described as a direct inhibitor here? Evidence is lacking. Lines 140-144: The description of “direct inhibitor” is unclear in these contexts of IFN-independent signaling (really just IRF3-independent), or physically direct interactions with components of the virus.*

**Response:** It was not our intention to suggest that ZAP or TRIM25 physically interact with the virus, but instead inhibit virus replication through a mechanism other than non-specific up-regulation of the IFN response through overexpression. This is an important distinction to make in these assays. However, we agree with the reviewer that the terminology could confuse readers. We have now removed “direct” from the abstract and text and have reworded the highlighted section and throughout the text.

*2) Figure 1/Supplemental Figure 1/Supplemental table 1: Figure 1 identifies a set of ISGs that reduce GFP expression (HCMV production) in both WT cells and IRF3KO cells. This suggests that several of the ISGs restrict HCMV in an IRF3-independent manner. While not explicitly stated, the ISG screen appears to have been performed as a single biological replicate. If the screen results are expected to serve as a resource to inspire future studies on HCMV pathogenesis and IFN functions, it is important that the authors perform at least one additional biological replicate. This will allow more confident identification of proteins that meet their criteria for ≥2-fold reductions in GFP expression (HCMV production) relative to the average GFP signal in both WT and IRF3 KO cells. Calculations of Z scores, standard deviation ranges, or p values (if biological replicates are performed) may be more informative than just fold-change assessment alone because these would better account for GFP signal variability. In line 149-150 and Suppl. Figure 1, ZAP expression did not inhibit primary replication in the screen (albeit with an n of 1?). Can the authors explain why? The authors should also include a legend for each supplemental table.*

**Response:** We have now included an additional biological repeat of the screen (Supplemental table 1). While there is expected variability between the screens as different arrayed library preps were used, the majority of top candidates show similar levels of inhibition, including ZAPS and TRIM25. Arrayed ISG screens are technically challenging and due to intrinsic variability caused by differences in lentivirus titre, they do not easily lend themselves to combining data sets from independent screens or analysis such as Z scores. However, they have proven highly effective for identifying ISGs, reflected by multiple publications based on single screens and careful validation of hits of interest. Moreover, these single screens have been a community resource, inspiring the identification of multiple antiviral interactions (by other groups) including one of the reports identifying Mx2’s anti-HIV-1 activity (PMID: 24055605) and multiple studies identifying SUN2’s antiretroviral activity (PMID: 26865710 and PMID: 28747499). To our knowledge this is the first and only screen of this type for HCMV. While the focus of this paper is the role of ZAP and TRIM25 in HCMV replication, we still believe the arrayed ISG screen is of value to the community. We have maintained the figures based on the initial screen as the virus production results for IRF3KO cells in the second screen were lower.

In the original primary screen, ZAP did inhibit virus replication, but did not result in a 50% reduction. This likely reflects a lower titre for the ZAP lentivirus in this screen, that results in less inhibition in the first screen, but still lead to substantial inhibition of virus production. However, we clearly show that ZAP overexpression inhibits HCMV replication through careful validation using titre matched stocks of lentivirus, demonstrating effective identification of host restriction factors. The main objective of screens is to identify potential hits of interest for further investigation.

*3) Figure 2 (and throughout): Figure 2 characterizes the impact of ZAP and TRIM25 on HCMV progeny production, demonstrating that they are restriction factors. Is there any comparison between endogenous and ectopic levels of ZAP expression in overexpression experiments? How do the ectopic levels relate to the levels during infection? Without this comparison it is very difficult to interpret how meaningful the overexpression phenotypes are.*

**Response:** We have now included a dilution series of ZAPS and TRIM25 lentivirus transduction comparing inhibition of virus and expression levels of the proteins (Supplemental Figure 2A and B).

*In Figure 2A-B, how do the authors explain the differences in relative GFP fluorescence when ZAPS is overexpressed in WT versus IRF3KO cells? Assuming statistical comparisons relative to the empty vector control, why did cGAS expression statistically significantly reduce GFP fluorescence relative to the empty vector control in IRF3KO cells?*

**Response:** Apologies, these observations had been addressed but were removed in a subsequent draft. The text has now been restored. “As expected, efficient cGAS inhibition is dependent on IRF3 expression, although inhibition was not completely rescued in IRF3 KO cells, suggesting cGAS may have antiviral activity that is independent of IRF3 signalling. There was also partial rescue of virus replication in IRF3 KO cells expressing ZAPS suggesting that some ZAPS inhibitory activity could depend on IRF3 signalling. However, virus replication was still significantly inhibited in wild type and IRF3 KO cells expressing ZAPS or TRIM25, corroborating the results from the screen and indicating that these ISGs inhibit HCMV virus replication in a manner independent of their role in the interferon pathway.”

This is further addressed in the discussion (line 451- 456).

*In Figure 2D-E, the data presented are not convincing enough to support the claim that endogenous ZAP has a meaningful impact on the virus. There is very little difference between depletion and the controls. Some differences appear to be well within error. An n of 2 is not sufficient here, should be repeated at least 3 times (full biological repeats). Differences are claimed despite the fact that error bars often overlap. ANOVA is not an appropriate statistical test here. Even a very crude analysis of target versus control at a single relevant time-point would be more meaningful.*

**Response**: We respectfully disagree. There is a clear statistical difference between control cells and knockdown cells. We would not expect a large increase in virus replication as replication is already occurring in fibroblast cells at high levels and in the case of ZAP, the virus has evasion mechanisms as discussed. Despite this, at 96 hours post infection there is an approximate one and half log increase in virus replication, with clear increases observed at four of the six time points measured. We have discussed this with our in-house statistician who assures us that ANOVA is an appropriate tool for this analysis. Whether the difference is biologically meaningful is inevitably somewhat subjective without in vivo experiments.

*The authors state that TRIM25 is required for ZAPS antiviral activity. They also state that siRNA depletion of TRIM25 reduces ZAPS expression during HCMV infection (Lines 292-293, Figure 8A) and that this occurs via differential ZAP splicing. Given these findings, one would expect that the effect of ZAPS depletion on HCMV progeny production would be phenocopied by TRIM25 depletion at the same times post-infection. Yet, HCMV progeny production at 72, 96, and 120 hpi (when ZAPS deletion appeared most effective in restricting HCMV) showed little to no difference in siTRIM25 cells compared to siNeg cells. The authors should explain this discrepancy.*

**Response**: While TRIM25 has been reported to be required for ZAP activity, we have not confirmed that TRIM25 is required for ZAP activity against HCMV. While the reviewer is correct that we show that knockdown of TRIM25 results in a failure in ZAPS induction, it also results in increased ZAPL expression, whereas the siRNA we used in figure 2 targets both ZAPS and ZAPL. Therefore, we would not expect identical results from knocking down ZAP and TRIM25. We have included additional text in the discussion to address this (line 537 - 540)

*4) Figures 4 and 5: Figures 4 and 5 identify a correlation between low ZAP and high IE2 levels in infected cells and demonstrate that IE1 levels are unaffected by ZAP expression. Line 222-224: If ZAPL expression shows the same inhibitory effects as ZAPS expression, then why do the authors believe that upregulation of the short form specifically is so important for restriction of the virus? Immunoblot levels of both ZAP isoforms appear to show that ZAPL is more abundant than ZAPS, further suggesting that both isoforms are capable of antagonizing IE2, pp52, and pp28 protein expression. This should be tested for ZAPL during HCMV infection as in Figure 5.*

**Response**: We initially focused on ZAPS as this is considered an ISG and is therefore included in the library, whereas ZAPL is considered constitutive rather than induced by IFN (in terms of transcript/protein abundance). However, as the reviewer points out, it is possible that both isoforms are relevant and indeed we believe that both ZAPS and ZAPL may play important roles during HCMV infection. Unfortunately, overexpression of ZAPL is toxic in primary fibroblast cells, making equivalent experiments impossible.

*Was there any attempt to reproduce these phenotypes? Quantification seems of little meaning without reproduction and error bars. These transfection data should also be done with increasing amounts of plasmid to strengthen the anticorrelation.*

**Response**: We have now included repeat western blot data and titration of ZAP plasmid showing clear anti-correlation between ZAPS expression and pp52.

*Is this restriction at the level of transcription or translation? Have they looked at mRNA levels? Figure 5 is similar in message to Figure 4 but includes HCMV infection instead of HCMV gene transfection and could really just be part of Figure 4.*

**Response**: We thank the reviewer for this suggestion and have now included data for RNA levels. Interestingly, we observe increased levels of RNA for IE1 and IE2 in particular. As explained in the text (Line 283 - 287) this may be an artefact of IE2 negatively regulating the CMV promoter in the expression plasmid. However, the results clearly show that loss of viral protein levels following expression of ZAP is not due to RNA degradation, indicating the effect of both ZAPS and ZAPL are post transcriptional.

*5) Figures 6 & 7, and the corresponding results sections: This section of the results aims to establish the effect of endogenous ZAP on viral replication. Despite the fact that ZAP can be knocked down by siRNA (Supplemental Figure 2), the ability of the virus to replicate in ZAP knockdown cells is not investigated further beyond the analysis of viral progeny production presented in Figure 2. Further experimentation to investigate the effect of ZAP knockdown on viral proteins and/or viral DNA replication would do a lot to substantiate the key claims. In addition, an alternate knockdown/knockout approach is crucial to account for the risks of off-target effects, artefacts, and transiency of siRNA-mediated knockdown over six-day long experiments. Unfortunately, the experiments reported in Figures 6 & 7 do very little to mitigate this.*

**Response**: We have now included western blot analysis of infected cells during siRNA knockdown (Figure 6B). There are relatively minor changes in IE gene expression with more obvious increases in pp52 and pp28 expression in ZAP knockdown cells. The western blot also shows effective knockdown of ZAP over the six-day period of the infection, which is consistent with our experience with siRNA knockdown in these cells. We use a pool of four siRNAs for these experiments. We have included deconvoluted data showing three of the four siRNAs from the pool result in increased HCMV replication, indicating the phenotype is highly unlikely to be due to off target effects (Supplemental figure 3).

*When considering the anti-correlation between ZAPS levels and viral protein levels, it is difficult to interpret what is the cause and what is the effect. The progress of virus infection is often heterologous at a cell to cell level. It is possible that the anti-correlation reflects more successful infection when ZAPS levels are low. However, it is also possible that ZAP levels are reduced as infection progresses (Figure 6A shows a very clear reduction in ZAPL as infection progresses, but also a more modest reduction in ZAPS). Presumably the antibody used for IF in Figure 6B stains both L and S isoforms? Would the cell-cell variation be diminished in ZAP knockdown cells? Additionally, as ZAP has previously been shown to bind CpG dinucleotides in viral RNA and target it for degradation, it would be interesting if the authors explored the binding of ZAPS to HCMV CpG dinucleotides, or assessed the mRNA levels of the observed ZAPS-antagonized HCMV gene products (e.g. pp52, pp28).*

**Response**: We agree that the data does not distinguish between the two different models and have now included text to acknowledge this (line 357 - 358). The potential of HCMV to reduce ZAP levels will require additional investigation. However, our data clearly shows that IE1 expression can occur in cells expressing high levels of ZAP, supporting our conclusion that reduced CpG levels in IE1 enables avoidance of targeting by ZAP. This is now strengthened by additional data showing increased CpG levels in IE1 allows ZAP dependent inhibition of expression (Figure 5). To determine whether ZAP binds CpGs in HCMV as the reviewer suggests, we are currently investigating binding of ZAP to viral transcripts through CLIP-seq studies. However, this represents a significant undertaking and as such falls out with the scope of this study.

*Lines 256-259 and Figure 6B: Contrary to what the authors state, it should be additionally noted that TB40/E-GFP can be certainly used for this microscopy experiment. Rabbit anti-ZAP primary antibody can be detected via an Alexa-fluor-555/568 (or 633/647) conjugated anti-rabbit secondary antibody. Mouse anti-IE1/IE2 primary antibody can be detected via an Alexa-fluor-633/647 (or 555/568) conjugated anti-mouse secondary antibody. This obviates the stated need to switch HCMV strains from TB40/E-GFP to AD169.
In Figure 7, cell sorting gives a population enriched for cells in which infection has progressed the furthest. The loss of ZAPS protein levels over time (Figure 7B) is even clearer in this advanced population. Further supporting the alternate hypothesis, that low ZAPS levels are the result of advanced infection, not the cause.*

**Response:** While we agree that this alternative approach would work for the IF, the use of AD169 seems reasonable as CpG levels of genes from both viruses are equivalent. Again, we completely agree with the reviewer that the experiment in figure 7 does not distinguish between the two proposed models and have now included text acknowledging this, as above. What it shows is that we do not observe advanced virus replication in cells that express high levels of ZAP and that IE1 is still expressed in populations of cells expressing high ZAP.

*6) Line 292, and Figure 8A: The authors claim that siTRIM25 results in increased viral proteins levels. This is not true of IE2, or of pp52, although pp28 does appear to be increased. Perhaps the most apparent difference between siTRIM25 and siNEG is the reduced IE1 at days 5/6. Can the authors offer an explanation for this?*

**Response**: We have now revised the text to indicate an increase in late viral gene expression (line 389). At this point we do not know why knockdown of TRIM25 results in reduced IE1 expression. This will require further investigation. However, the main focus of this figure is the effect of TRIM25 knockdown of ZAPS and ZAPL levels which is very clear.

*7) Figures 8C-F: The authors show that TRIM25 impacts ZAPS protein levels, suggesting that it mediates differential splicing of ZAP. The extent to which ZAP RNA levels are induced (uninfected to infected, or non-treated to IFN treated) is not apparent the way the data is presented. Were these figures generated from a single experiment? If not they should be repeated to show the difference between siNEG and siTRIM25 in relation to the magnitude of induction. Are all of these differences believed to be biologically meaningful (for example, 8C, ZAPS). This approach was considered appropriate in supplemental figure 6, so why not here? Does the n=2 represent full biological replicates? Given the identical exon sequences of ZAPS and ZAPL up to the 699 residues of ZAPS, how are the authors differentiating ZAPS transcript abundance from ZAPL in the “ZAPS” columns? RT-qPCR analysis of any region within ZAPS may also amplify ZAPL.*

**Response**: We agree and have reformatted the data accordingly. The results are based on two biological repeats and we have now included an additional time point for infected cells. We note that the decrease of ZAPS in infected cells falls just outside significance (P value = 0.06 and 0.07 respectively) but suggest that the trend at two time points is clear. Yes, we believe the clear pattern of increased ZAPL and corresponding decreased ZAPS RNA levels are biologically meaningful, especially when considered along with the western blot analysis which shows a clear loss in ZAPS induction and increased ZAPL expression. This is likely to have important implications for how TRIM25 functions in relation to ZAP. ZAPS and ZAPL have independent 3’UTRs allowing differentiation by PCR. We have now clarified this in the text (line 398 - 399).

***Reviewer #2:***

*1. The ultimate experiment to further support the authors’ main conclusion would be to increase the CpG content in the IE1 coding region to test whether this enhances inhibition by ZAP. Ideally, this experiment would be done in the context of the hCMV genome, but that would involve the generation of informative mutant viruses which might be difficult (in part due to potential side effects from mutagenesis within the highly complex major IE region of the viral genome, as mentioned at the end of the Discussion). However, similar experiments could be done using transfected plasmids as in Fig 4. Such experiments would greatly enhance the study.*

**Response**: We thank the reviewer for the suggestion. We have now included data for an IE1 construct with increased CpG content. As predicted this construct is susceptible to ZAP inhibition.

*2. The data on IE2 are less consistent than those on IE1. Although CpG content does not appear to be significantly suppressed in hCMV IE2 (Fig 3), there is little reduction of IE2 (relative to pp52) by ZAP expression in transfected cells (Fig 4). Then again, IE2 levels are quite dramatically reduced by ZAP expression during hCMV infection (Fig 5). The authors should try to explain these somewhat discordant findings.*

**Response**: Following additional repeats of the plasmid co-expression experiments we do see a clear reduction in IE2 protein levels, consistent with the intermediate levels of CpG content in the IE2 gene. We predict that the more substantial reduction in IE2 protein following over expression of ZAPS in the context of HCMV infection is unlikely to be a direct result of ZAP targeting IE2 transcript. Our previously published observations, as well as others, indicate that IE2 is initially expressed at low levels, before increasing substantially after the onset of DNA amplification. Our data suggests that inhibition of HCMV by ZAPS overexpression, occurs prior to the exponential increase in IE2 expression. We have now included text to reflect this.

*3. By definition, IE proteins are expressed at very early times during hCMV infection. However, IE2 does not seem to accumulate to significant levels before day three post infection (e.g. Fig 5, 6 and 8), possibly suggesting that the MOI may be lower than specified. Why do the authors think IE2 comes up that late in their system?*

**Response**: Similar to above, in previous publications we observe low levels of expression of IE2 as early as 6 hours post infection, but substantial increase in expression of IE2 occurs 24-48 hours post infection. We agree the kinetics of IE2 expression are likely related to MOI. When we increase the MOI to five or higher we can detect IE2 expression by western blot as early as 24 hours post infection. Based on GFP expression we are confident that we are infecting the vast majority, if not all cells at the MOI used.

***Reviewer #3:***

*Fig 4 clearly shows that ZAPS and ZAPL both reduce protein levels of p52 but not IE1, but what about mRNA levels for these viral orfs? Since the authors note in the discussion that previous studies of RNA viruses have suggested distinct mechanisms of action for ZAP, which may or may not include RNA degradation, this should be determined directly for the case of HCMV ie1 vs p52 transcripts. Additionally, as they have shown some very interesting differences in the regulation of ZAPS and ZAPL in various experimental scenarios, these analyses could provide significant insight into how these two differentially spliced transcripts operate.*

**Response**: We thank the reviewer for the suggestion. We have now included RNA levels for these experiments, with RNA harvested from the same cells used to generate repeats of the Western blot experiments to allow direct comparisons. Interestingly, despite significant decreases in pp52 protein levels, we do not observe a concurrent reduction in RNA levels, indicating that ZAPS and ZAPL are functioning in a post transcriptional manner. Further investigation will be required to define the precise mechanism of inhibition by ZAP.

*The authors raise an interesting hypothesis in their discussion that the relative large differences in the levels of CpG throughout the alpha, beta and gamma herpesvirus genomes might be due to latency establishment in distinct cell types. It is quite likely that CMV establishes latency in endothelial cells in addition to hematopoietic cells, and little evidence suggest fibroblasts are true latent reservoirs. As the authors have performed the vast majority of their studies with the TB40/E strain, this provides them an opportunity to assess whether ZAP/TRIM25 function in this cell type to suppress non-IE gene expression similar to fibroblasts. So many questions exist in this field regarding whether a particular host innate defense mechanism operates similarly in distinct cell types. Even just assessing whether ZAP S and L are expressed in human endothelial cells (e.g. HUVEC), are inducible via IFN-I signaling and if their overexpression shows similar inhibition of HCMV replication would increase the impact of their study significantly.*

**Response**: We thank the reviewer for the suggestion and agree that distinct roles of the innate immune response in different cell types and during acute versus latent infection is certainly an area of interest for us going forward. The dynamic range of infection in endothelial cells and epithelial cells is much lower than fibroblast cells due to restricted replication of the virus. However, we have now included data showing that lentivirus transduction with ZAPS and TRIM25 results in substantial inhibition of HCMV in both epithelial cells and endothelial cells. We are actively investigating the ISG response to HCMV in these cell types, including further characterisation of ZAP and TRIM25, however, this will require a significant amount of work to fully characterise and as such falls outside the scope of this study.

**Part III – Minor Issues: Editorial and Data Presentation Modifications**
Please use this section for editorial suggestions as well as relatively minor modifications of existing data that would enhance clarity.

***Reviewer #1:***

*1) Introduction: Sentence beginning on line 68 (“RNA and small DNA…”) should be split into 2 sentences for clarity.

2) Line 153: “dependent” is misspelled.

3) Line 170: “efficient cGAS inhibition” could have mixed interpretations so would suggest re-wording.

4) Line 294-297: Please cite the literature.*

**Response**: All corrections have been made

***Reviewer #2:***

*4. The title and parts of the main text (e.g. lines 200-201) suggest that there is more than one major IE gene and that all of them are affected by CpG suppression, which does not seem to be the case.*

**Response**: We agree and have changed the title and where referencing HCMV. For line 200-201 we refer to beta-herpesviruses which do show suppression of more than one MIE gene.

*5. I do not agree based on the presented data that “ZAP and TRIM25 are rapidly reduced” during hCMV infection (line 34). They are reduced, but not rapidly and maybe not efficiently enough to explain the normal CpG content in viral genes other than IE1. Can the authors speculate about hCMV antagonists of ZAP/TRIM25?*

**Response**: We agree and have now reworded this sentence. We have also added text to acknowledge the potential of ZAP/TRIM25 antagonists (line 498 - 499).

*6. Combine sentence in lines 90-91 with previous paragraph.*

**Response**: This change has been made

*7. Provide reference for “Individuals with mutations in key IFN signalling genes are lethally susceptible to HCMV infections…” (lines 102-103).*

**Response**: references have now been included

*8. Clarify that IRF3, IRF7 and NFKB typically activate IFN genes first, and that ISGs are usually activated as a consequence of that (line 112).*

**Response**: This has now been clarified

9. Specify (in figure legend) or remove horizontal red line in Fig 1D.

**Response**: The line has now been removed.

*10. Correct “These genes are the first viral transcripts to be expressed…” (line 201), since genes are not transcripts.*

**Response**: This has been corrected

*11. Remove full stops from subheadings (line 211 and 244).*

**Response**: These have been removed

*12. All abbreviations used in the figures, including “EMP” and “UN”, should be defined in the figure legends.*

**Response**: All abbreviations have now been defined.

*13. Discuss whether CpG suppression extends to the hCMV major IE promoter-enhancer (and other non-coding sequences) or if it is restricted to the open reading frames. What could this mean, if CpG suppression is not limited to open reading frames?*

**Response**: This is an interesting question. The upstream region containing the MIE promoter, not including regions that overlap with predicted ORFs (527 bps) has a CpG ratio of 0.71, so doesn’t show the same level of suppression as IE1 exon 4. Extending the sequence to 1000 bps upstream of the MIE transcriptional start site also shows a ratio of 0.71. We have also used a sliding window to scan CpG levels across the whole genome and IE1 exon 4 is the only extended region of suppressed CpG ratio within the virus. Text has been included in the discussion (line 479 - 481).

*14. Replace “student T test” (e.g. legend to Fig 8) with “Student’s t-test”.*

**Response:** Correction made.

*15. Reference 47 is about the role of ZAP in alphavirus, not alpha-herpesvirus (HSV-1) replication (line 378-380). Might the fact that alpha-herpesviruses replicate more quickly (express ZAP/IFN antagonists earlier?) than beta-herpesviruses explain partly the differences in CpG suppression?*

**Response:** Thank you for pointing this out. The correct reference has now been included. Yes, this is certainly a possibility. Also, HSV-1 expresses potent host shut-off activity which may also play a role. A specific ZAP inhibition mechanism cannot be ruled out either.

***Reviewer #3:***

*The data in Fig 6 showing that at the individual cell level, cells expressing higher levels of ZAPS show reduced IE2 expression, but not IE1, is a very elegant result.

Data shown in Fig. 8 indicating that TRIM25 can regulate the differential splicing of ZAP between its long and short isoforms is the first example of TRIM family proteins operating at this level, which is highly interesting.

The paper is very clearly written, it was an enjoyable read.*

**Response:** We appreciate the positive feedback.
